# Direct Molecular Diagnosis of Aspergillosis and CYP51A Profiling from Respiratory Samples of French Patients

**DOI:** 10.3389/fmicb.2016.01164

**Published:** 2016-07-29

**Authors:** Yanan Zhao, Cécile Garnaud, Marie-Pierre Brenier-Pinchart, Anne Thiébaut-Bertrand, Christel Saint-Raymond, Boubou Camara, Rebecca Hamidfar, Odile Cognet, Danièle Maubon, Muriel Cornet, David S. Perlin

**Affiliations:** ^1^New Jersey Medical School, Public Health Research Institute, Rutgers Biomedical and Health SciencesNewark, NJ, USA; ^2^Laboratoire de Parasitologie-Mycologie, Institut de Biologie et Pathologie, Centre Hospitalier Universitaire Grenoble AlpesGrenoble, France; ^3^Laboratoire TIMC-IMAG-TheREx, UMR 5525 Centre National de la Recherche Scientifique, Université Grenoble AlpesGrenoble, France; ^4^Institut Albert Bonniot, Centre National de la Recherche Scientifique UMR 5309, Institut National de la Santé et de la Recherche Médicale U1209, Université Grenoble-AlpesGrenoble, France; ^5^Clinique Universitaire d'Hématologie, Centre Hospitalier Universitaire Grenoble AlpesGrenoble, France; ^6^Clinique Universitaire de Pneumologie, Centre Hospitalier Universitaire Grenoble AlpesGrenoble, France; ^7^Réanimation Médicale, Centre Hospitalier Universitaire Grenoble AlpesGrenoble, France

**Keywords:** *Aspergillus fumigatus*, azole resistance, respiratory samples, French patients, CYP51A, molecular diagnostics

## Abstract

**Background:** Microbiological diagnosis of aspergillosis and triazole resistance is limited by poor culture yield. To better estimate this shortcoming, we compared culture and molecular detection of *A. fumigatus* in respiratory samples from French patients at risk for aspergillosis.

**Methods:** A total of 97 respiratory samples including bronchoalveolar lavages (BAL), bronchial aspirates (BA), tracheal aspirates, sputa, pleural fluids, and lung biopsy were collected from 33 patients having invasive aspergillosis (*n* = 12), chronic pulmonary aspergillosis (*n* = 3), allergic bronchopulmonary aspergillosis (*n* = 7), or colonization (*n* = 11) and 28 controls. Each specimen was evaluated by culture, pan-*Aspergillus* qPCR, and CYP51A PCR and sequencing.

**Results:** One *A. flavus* and 19 *A. fumigatus* with one multiazole resistant strain (5.3%) were cultured from 20 samples. Culture positivity was 62.5, 75, 42.9, and 15.8% in ABPA, CPA, IA, and colonized patients, respectively. *Aspergillus* detection rate was significantly higher by pan-*Aspergillus* qPCR than by culture in IA (90.5 vs. 42.9%; *P* < 0.05) and colonization group (73.7 vs. 15.8%; *P* < 0.05). The CYP51A PCR found one TR_34_/L98H along with 5 novel *cyp51A* mutations (4 non-synonymous and 1 promoter mutations), yet no association can be established currently between these novel mutations and azole resistance. The analysis of 11 matched pairs of BA and BAL samples found that 9/11 BA carried greater fungal load than BAL and CYP51A detection was more sensitive in BA than in BAL.

**Conclusion:** Direct molecular detection of *Aspergillus* spp. and azole resistance markers are useful adjunct tools for comprehensive aspergillosis diagnosis. The observed superior diagnostic value of BAs to BAL fluids warrants more in-depth study.

## Introduction

*Aspergillus* spp., especially *Aspergillus fumigatus*, are the main causes of a wide spectrum of diseases including invasive aspergillosis (IA), chronic pulmonary aspergillosis and allergic syndromes. Highly active triazoles except fluconazole are recommended as the first-line therapy to treat aspergillosis, but their effectiveness is challenged by the emergence of drug resistance (Verweij et al., 2015; Denning et al., 2016). Since the late 2000s, when two distinct routes of resistance development (environmental exposure to azole fungicides and long-term azole therapy) were reported (Verweij et al., 2007; Howard et al., 2009), a number of national or local hospital/medical center-based surveys have been carried out to assess local or regional epidemiology of azole resistance in *A. fumigatus* (Lockhart et al., 2011; Chowdhary et al., 2015; Van Der Linden et al., 2015; Vermeulen et al., 2015; Le Pape et al., 2016). These epidemiological studies revealed striking variance on the prevalence of resistance in different geographical areas or subpopulations, for example, a recent national survey in the Netherlands reported that azole resistance rates at the hospital level varied between 5 and 10%, although rates up to 30% were reported in high-risk wards (Lestrade et al., 2016). One problem with assessing prevalence of resistance is noted as the recovery of *A. fumigatus* in culture is generally low and may vary considerably among different patient groups. This outcome indicates that in culture-negative patients, the presence of azole resistance will be missed (Van Der Linden et al., 2016).

In fact, *A. fumigatus* is notorious for its low culturability from infection sites, especially in chronic infection patients as well as neutropenic IA patients (Denning et al., 2011; Bergeron et al., 2012). By using a highly sensitive and specific PCR method, it was previously shown that the resistance-associated *cyp51A* mutation was found in as high as 55% of non-culturable but *Aspergillus* PCR positive respiratory samples (Denning et al., 2011). Although such high resistance rate was not observed in subsequent studies from different patient populations, direct detection of *A. fumigatus* and azole resistance markers from bronchoalveolar lavage (BAL) fluid did demonstrate cryptic azole resistance that may be missed by culture-based diagnosis (Zhao et al., 2013).

In France, both high and low prevalence of azole resistance in *A. fumigatus* clinical isolates have been reported from different populations and locations (Alanio et al., 2011, 2016; Burgel et al., 2012; Morio et al., 2012; Choukri et al., 2015), but no study has assessed diagnostic value of direct detection of *Aspergillus* spp. and azole resistance marker from primary samples in populations at high risk for *Aspergillus* infections. Herein, we compared diagnostic performance of culture and molecular detection of *Aspergillus* spp. and *A. fumigatus* CYP51A profiling in respiratory samples from patients with various underlying diseases at one medical center in France.

## Materials and methods

### Patient characteristics and sample collection

A total of 97 respiratory samples (including BAL, BA, tracheal aspirates, sputa, pleural fluids, and lung biopsy) from 33 patients and 28 controls collected at the Grenoble Alpes University Hospital in Grenoble, France, between October 2013 and July 2014 were included in the study. Allergic bronchopulmonary aspergillosis (ABPA), chronic pulmonary aspergillosis (CPA), and IA were defined in accordance with the classifications established by the international committees of experts available at the time of the study (Denning et al., 2003; Stevens et al., 2003; De Pauw et al., 2008; Smith and Denning, 2011; Garnaud et al., 2012; Agarwal et al., 2013; Schweer et al., 2014). Colonization was defined as a culture (or a previous history) that yielded *Aspergillus* spp. from respiratory specimens in patients who did not match the criteria for IA, CPA or ABPA diagnosis and had no major respiratory symptoms. Control patients were defined as having pulmonary symptoms and in whom the diagnosis of *Aspergillus* infection or colonization was ruled out. The underlying diseases are listed in Table 1. All specimens were aliquoted upon collection in a biosafety cabinet and subjected to culture on Sabouraud-Chloramphenicol tubes and Candida ID2 plates (bioMérieux, Marcy l'Étoile, France), pan-*Aspergillus* real-time PCR, and CYP51A PCR and sequencing. Aliquots for PCR testing were stored frozen at −80°C until process. According to the clinical evaluation and the requests from the physician, 18 samples from 13 patients (3 controls, 1 colonization, 1 CPA, and 8 IA) were also tested for galactomannan (GM) with the Platelia® Aspergillus assay (BioRad, Marnes-la-Coquette, France).

**Table 1:** **Underlying conditions of the patients and controls**.

**Clinical diagnosis**	**No. of patients (no. of specimens) with underlying conditions**						**Total**
	**CF**	**CF + SOT**	**SOT**	**HAEM**	**PULM**	**Other**	
ABPA	6 (6)	0	0	0	1 (2)	0	7 (8)
CPA	0	0	1 (1)	0	2 (3)	0	3 (4)
IA	1 (1)	0	1 (2)	7 (15)	2 (2)	1 (1)	12 (21)
Colonization	1 (1)	2 (5)	5 (8)	2 (4)	1 (1)	0	11 (19)
Control	3 (4)	1 (1)	6 (11)	7 (11)	11 (18)	0	28 (45)
Total	11 (12)	3 (6)	13 (22)	16 (30)	17 (26)	1 (1)	61 (97)

*CF, cystic fibrosis; SOT, solid organ transplantation; HAEM, hematological malignancies;PULM, pulmonary disorders other than CF; Other, digestive surgery*.

### Susceptibility testing

Following species identification, all *A. fumigatus* isolates were assessed by Etest® (bioMérieux) and the resistant isolate was subjected to microdilution susceptibility testing for itraconazole, voriconazole and posaconazole following the Clinical Laboratory and Standards Institute protocol M38-A2 (CLSI, 2008).

### DNA extraction and CYP51A profiling

Excluding sputa and one lung biopsy, all fluid samples (1 ml when available, otherwise the entire volume) were centrifuged at 16000 g for 10 min at 4°C. Pellets, sputum and lung biopsy samples were processed with DNA extraction, using MasterPure™ Yeast DNA Purification kit (Epicentre Biotechnologies, Madison, WI, USA), as described previously (Zhao et al., 2013). A pan-*Aspergillus* real-time PCR along with nested PCR amplification of the *A. fumigatus* CYP51A ORF and promoter region, and DNA sequencing of PCR amplicons were performed blinded on all samples, as described previously (Zhao et al., 2013).

### Statistical analysis

Fisher's exact test was used to compare detection rate of culture and PCR. A *P* < 0.05 was considered statistically significant.

### Ethics statement

This study was approved by Rutgers Health Sciences Institutional Review Board (IRB).

## Results

### Detection of *Aspergillus* spp. from respiratory samples

A total of 20 samples (7 BA, 2 BAL, 9 sputa, and 2 tracheal aspirates) were culture positive for *Aspergillus spp*., including 19 *A. fumigatus* and 1 *A. flavus*. *Aspergillus* culture positivity was 62.5, 75, 42.9, and 15.8% in samples from ABPA, CPA, IA, and colonized patients, respectively. No *Aspergillus* was isolated from control samples, but one sample was culture-positive for *Exophiala spp*. and one was culture-positive for *Penicillium spp*. (Table 2). In comparison, *Aspergillus* detection rate was generally higher in all patient groups by pan-*Aspergillus* real-time PCR than by culture. In particular, PCR positivity was significantly higher than culture positivity in the IA group (90.5 vs. 42.9%; *P* < 0.05). The same significant difference was observed in the group of colonized patients (73.7 vs. 15.8%; *P* < 0.05). Only 1/45 (2.2%) control samples were pan-*Aspergillus* PCR positive. Among 18 specimens (8 BAL, 5 BA, 2 tracheal aspirate, 1 pleural fluid, 1 sputum, and 1 lung biopsy) available for GM evaluation, 10/11 GM-positive and 2/7 GM-negative samples were pan-*Aspergillus* PCR positive with culture-positive for one *A. fumigatus* and one *A. flavus*.

**Table 2:** **Culture, pan-***Aspergillus*** PCR, and CYP51A PCR detection in different patient groups**.

**Clinical diagnosis**	**Culture**				**pan-*****Aspergillus*** **PCR**			**CYP51A PCR**		
	***Aspergillus spp*.^a^**	**Non-*Aspergillus spp***.	**neg**.	**Total (%)^e^**	**Pos**.	**Neg**.	**Total (%)**	**Pos**.	**Neg**.	**Total (%)**
ABPA	5	1^b^	2	8 (62.5)	6	2	8 (75)	6	2	8 (75)
CPA	3	0	1	4 (75)	4	0	4 (100)	4	0	4 (100)
IA	9	0	12	21 (42.9)	19	2	21 (90.5)^f^	16	5	21 (76.2)
Colonization	3	1^c^	15	19 (15.8)	14	5	19 (73.7)^f^	12	7	19 (63.2)
Control	0	2^d^	43	45	1	44	45 (2.2)	0	45	45
Total	20	4	73	97	44	53	97	38	59	97

a *Except one isolate from an IA patient is A. flavus, all isolates are A. fumigatus*.

b *Scedosporium spp*.

c *Penicillium spp*.

d *Non-Aspergillus spp. include 1 Exophiala spp., and 1 Penicillium spp*.

e *% is Aspergillus spp. culture positivity*.

f *PCR detection positivity significantly higher than culture, P = 0.0025 for IA group, and P = 0.0008 for colonization group*.

### Azole resistance profile

According to susceptibility testing results, all *A. fumigatus* isolates were susceptible to all three tested azole drugs except one from a colonized patient demonstrating a multiazole resistant pattern (MIC results itraconazole 16 mg/L; posaconazole 1 mg/L; voriconazole 4 mg/L). This patient received daily posaconazole (800 mg/d) treatment for one year prior to the sampling. The resistance rate estimated from *A. fumigatus* culture was 1/19 (5.3%). When *A. fumigatus* CYP51A PCR was applied directly to respiratory samples, 38 of 44 pan-*Aspergillus* PCR positive samples were successfully amplified. Among the six samples that failed to amplify in CYP51A PCR, one was *A. flavus* culture-positive, one was *Penicillium* culture-positive, and another culture-negative sample was positive in species-specific real-time PCR for *A. flavus* but not *A. fumigatus* (data not shown). The remaining 3 pan-*Aspergillus* PCR positive but CYP51A PCR negative samples had Ct values of 34.3, 34.8, and 35.9 in pan-*Aspergillus* detection, respectively, suggesting a low amount of *Aspergillus* DNA in these samples. Non-synonymous mutations (Table 3) were observed in 5/38 (13.2%) CYP51A PCR positive samples, and 3 of which were from culture negative specimens. Except for one TR_34_/L98H found in a sputum sample that was culture-positive with the multiazole resistant profile, other mutations causing amino acid substitutions in the cyp51A protein were novel. In addition to the patient with the TR_34_/L98H mutation, previous azole therapy was found in two other patients showing CYP51A mutated *Aspergillus* (Table 3).

**Table 3:** *****A. fumigatus*** CYP51A mutations detected directly from respiratory samples**.

**Specimen**	**Patient category**	**Clinical diagnosis**	**Culture/azole susceptibility**	Mutations^*^	**Prior azole therapy dosage and duration**
Sputum	SOT	Colonization	*A. fumigatus*/ITC = 16 mg/L, POS =1 mg/L, VRC = 4 mg/L	**TR_34_/L98H**	POS 800 mg/d for 1 year
BAL	SOT	Colonization	Negative	**I217T**	No
BA	HAEM	Colonization	Negative	**R355H**	No
BA	HAEM	Colonization	Negative	**L399F**	No
Sputum	CF	ABPA	*A. fumigatus*/susceptible	**I354V**	No
BA	PULM	CPA	*A. fumigatus*/susceptible	promoter -129 T to C	VRC 400 mg/d for 3 months until 3 months before sampling
BAL	HAEM	IA	Negative	D70D; T140T	No
BA	HAEM	IA	Negative	L77L	No
BA	PULM	ABPA	Negative	R65R	No
BAL	SOT	Control	Negative	E356E	VRC 400 mg/d for 2 years

*ITC, itraconazole; POS, posaconazole; VRC, voriconazole*.

* *Non-synonymous mutations are bolded*.

### Respiratory sample type impact on *Aspergillus* detection

To investigate the diagnostic value of different respiratory sample types for both *Aspergillus* and azole resistance detection, we analyzed 11 matched pairs of BAL and bronchial aspirate (BA) samples collected during the same bronchoscopy exam, from 11 patients with different underlying diseases and clinical diagnosis (Table 4). Interestingly, 9 out of 11 BA samples (from patient A to I) had an average of 4.7 lower pan-*Aspergillus* Ct value than their matched BAL samples, indicating a greater load of *Aspergillus* in BA than in BAL, regardless of underlying diseases or infection status. In particular, while robust signals were detected in BAs from patient A and patient I, matched BAL fluids from these two patients failed to generate positive amplicon signals. As a consequence of such *Aspergillus* load difference, while CYP51A sequence was available from both sample types for four patients (patients B, G, H, and J), in six patients (patients A, C, D, E, F, and I) it was only detectable from BA samples. Two non-synonymous CYP51A mutations were found, one (I217T) was in the BAL fluid from patient B, and the other (L399F) was in the BA from patient D. Patient J had very close *Aspergillus* burden measurement [ΔCt_(BAL−BA)_ = −0.6] in the matched set of BA and BAL, but only the BA was culture positive for a susceptible *A. fumigatus*. The only exception to the general observation that BA tends to have greater fungal load resulting in higher *Aspergillus* and resistance marker detection positivity was patient K, who had a positive pan-*Aspergillus* PCR result with a Ct of 34.1, as well as positive CYP51A PCR detection, in his BAL, but the matched BA was negative in all tests. Regarding the culture results, no difference could be drawn between BAL and BA expect for patient J.

**Table 4:** *****Aspergillus*** and azole resistance profile in matched pairs of BA and BAL samples**.

**Patient**	**Specimen**	**Underlying disease**	**Clinical diagnosis**	**Culture/azole susceptibility**	**Pan-*Aspergillus* PCR**	**Ct**	ΔCt _(*BAL*−*BA*)_^*^	**CYP51A PCR**	**Mutations**
A	BA	CF+SOT	Colonization	Negative	Pos.	31.8		Pos.	None
	BAL	CF+SOT	Colonization	Negative	Neg.	No Ct	8.2	Neg.	NA
B	BA	SOT	Colonization	Negative	Pos.	31.7		Pos.	None
	BAL	SOT	Colonization	Negative	Pos.	33.4	1.7	Pos.	I217T
C	BA	HAEM	IA	Negative	Pos.	28.4		Pos.	None
	BAL	HAEM	IA	Negative	Pos.	35.9	7.5	Neg.	NA
D	BA	HAEM	Colonization	Negative	Pos.	33.4		Pos.	L399F
	BAL	HAEM	Colonization	Negative	Neg.	36.2	2.8	Neg.	NA
E	BA	CF+SOT	Colonization	*Penicillium spp*.	Pos.	30.6		Pos.	None
	BAL	CF+SOT	Colonization	Negative	Pos.	34.8	4.2	Neg.	NA
F	BA	SOT	Colonization	Negative	Pos.	33.9		Pos.	None
	BAL	SOT	colonization	Negative	Neg.	36.2	2.3	Neg.	NA
G	BA	SOT	IA	*A. fumigatus*/susceptible	Pos.	24.9		Pos.	None
	BAL	SOT	IA	*A. fumigatus*/susceptible	Pos.	27.1	2.3	Pos.	None
H	BA	HAEM	IA	*A. fumigatus*/susceptible	Pos.	29.6		Pos.	None
	BAL	HAEM	IA	*A. fumigatus*/susceptible	Pos.	34.4	4.9	Pos.	None
I	BA	PULM	ABPA	Negative	Pos.	31.4		Pos.	R65R
	BAL	PULM	ABPA	Negative	Neg.	No Ct	8.7	Neg.	NA
J	BA	HAEM	IA	*A. fumigatus*/susceptible	Pos.	32.4		Pos.	None
	BAL	HAEM	IA	Negative	Pos.	31.8	−0.6	Pos.	None
K	BA	HAEM	IA	Negative	Neg.	No Ct		Neg.	NA
	BAL	HAEM	IA	Negative	Pos.	34.1	−5.9	Pos.	None

*NA, not applicable*.

* *A Ct-value of 40 was used to calculate Ct difference between BA and BAL samples when no Ct was reported for pan-Aspergillus PCR*.

## Discussion

We have previously demonstrated the use of pan-*Aspergillus* PCR to uncover non-culturable *A. fumigatus* from respiratory samples (Denning and Perlin, 2011; Zhao et al., 2013). Consistently, in the present study application of pan-*Aspergillus* PCR resulted in higher detection rate than culture in all patient groups, particularly IA and colonized patients. Previously, the relatively high positivity (>30%) of *Aspergillus* PCR in control group (normal healthy volunteer or GM-negative BAL) was seen with small number of control samples in both studies. Therefore, we have increased the number of control samples in the current study, where 45/97 (46.4%) samples are considered as “*Aspergillus*-free” according to clinical diagnosis and microbiological laboratory tests. Except one positive result from one *Penicillium* culture-positive sample expected due to the cross-activity with *Penicillium spp*. of the assay, the PCR positivity in control group was 1/45 (2.2%) demonstrating excellent specificity of the test. This result further supports the notion of implementing PCR to facilitate rapid diagnosis of *Aspergillus* infections.

BAL fluid is the most commonly used respiratory sample type for the diagnosis of aspergillosis (Nguyen et al., 2007; Johnson et al., 2015). Yet, the diagnostic value of other respiratory sample types has not been fully defined. In the present study, BAL samples were collected from the distal pulmonary tract following instillation of a large volume of saline, about 100 ml in three aliquots. Bronchial aspirates were performed during the same bronchoscopy before BAL and after the end of BAL collection. Therefore, compared to BAL, BA is much less diluted and contain more mucous. In this study, we analyzed 11 matched pairs of BA and BAL samples from 11 patients with different underlying diseases and clinical diagnosis. Interestingly, a significantly higher *Aspergillus* load and resistance marker detection rate were observed in the majority of BAs than in their matched BALs. This result is consistent with two recent reports, where respiratory samples (bronchial wash or bronchial secretion) collected in a similar way demonstrated equal or even higher diagnostic yield compared to BAL fluids (Escribano et al., 2015; Taremi et al., 2015). In addition, it confirms a previous French study, which already suggested the superiority of the BA in the setting of IA diagnosis in hematological patients (Bergeron et al., 2012).

Although the prevalence of azole resistance in *A. fumigatus* has been investigated recently in diverse populations in France (Alanio et al., 2011, 2016; Burgel et al., 2012; Morio et al., 2012; Choukri et al., 2015), the true frequency of resistance remains largely unknown due to following reasons. First, the prevalence reported from previous studies was based on susceptibility testing of clinical *A. fumigat*us isolates, representing <50% of infections caused by this organism, even in confirmed aspergillosis cases (Reichenberger et al., 1999; Nguyen et al., 2011; Arendrup et al., 2012). Therefore, the presence of azole resistance in non-culturable *A. fumigatus* was missed by the culture-based assessment. Second, no permanent surveillance system based on sensitive *Aspergillus* azole resistance detection methods exist in France and the prevalence of resistance may differ considerably from each other depending on location of studied hospitals and patients' underlying diseases (Alanio et al., 2016), hence data obtained from certain sub-populations may not be representative for other populations with different underlying diseases or from different geographical areas. In the present study 5.3% (1/19) of *A. fumigatus* isolates displayed an azole resistance phenotype, lower than 8% in one CF population (Morio et al., 2012) but higher than 1.1% in hematological patients or 1.8% in unselected patients in France (Alanio et al., 2011). Considering the mixed composition of patients in the current study, this result was somewhat expected, and also supports the comment made by van der Linden et al. about the need to determine azole resistance frequency at the hospital level and within different patient groups (Van Der Linden et al., 2016).

In the present study, direct CYP51A detection enabled azole resistance profiling in 19 non-culturable or *A. fumigatus* culture-negative specimens in addition to other 19 *A. fumigatus* culture-positive samples, and one TR_34_/L98H along with 5 novel *cyp51A* mutations (4 non-synonymous mutations and 1 promoter mutation) were identified. Among the novel mutations, the I217T alteration detected from a culture-negative BAL may be of note, because previous work on three-dimension model of 14α-demethylase and related deduction has indicated that I217 is predicted to have direct interactions with either the substrate or triazole antifungal drugs (Ji et al., 2000; Mellado et al., 2004). Other novel mutations could possibly be natural polymorphisms. Nevertheless, whether or not these mutations are associated with azole resistance needs further evaluation. Compared to our previous findings in the UK patient population (Denning et al., 2011), direct CYP51A profiling has limited additive value to azole resistance diagnosis, which is largely due to the very low resistance rate in this particular population. No clear relation between prior azole exposure and *cyp51A* mutation could be determined from our study since only three patients (out of the 10 harboring mutated *cyp51A*) had received azole therapy before. The patient who had the typical environment-acquired TR_34_/L98H detected was treated with posaconazole for a year prior to sampling, therefore, it is difficult to tell the source of this mutation in this particular patient.

In summary, our study confirmed the advantage of molecular methods over culture on diagnosis of aspergillosis. Direct detection of CYP51A from primary samples is a faster and reliable azole resistance diagnostic method especially for culture negative samples, although its value may have been limited in this particular patient population with very low resistance rate. The observed superior diagnostic value of BA to BAL fluids warrants additional in-depth studies.

## Author contributions

CG, MB, AT, CS, BC, RH, OC, DM, and MC obtained clinical samples and provided clinical background information. YZ performed all molecular tests and resistant strain susceptibility test. YZ, DP, and MC designed the study, analyzed data, and wrote the paper. All authors discussed the results, commented on the manuscript, and agreed to be accountable for all aspects of the work in ensuring that questions related to the accuracy or integrity of any part of the work are appropriately investigated and resolved.

### Conflict of interest statement

DP has received support from the US National Institute of Allergy and Infectious Diseases and has received support from Cidara, Astellas, Matinas and Merck, and participates in consult panels for these companies. YZ has received research support from Merck. CG has received travel grants from MSD. DM has received travel grants from Pfizer. AT participates the consultant board for Pfizer, GILEAD, Bristol-Myers Squibb. MC has received travel grants from Gilead, Pfizer and Merck and received remuneration for talks on behalf of Pfizer. All other authors declare that the research was conducted in the absence of any commercial or financial relationships that could be construed as a potential conflict of interest.
